# Chronic cough and obstructive sleep apnoea in a sleep laboratory-based pulmonary practice

**DOI:** 10.1186/1745-9974-9-24

**Published:** 2013-11-05

**Authors:** Tsai-Yu Wang, Yu-Lun Lo, Wen-Te Liu, Shu-Min Lin, Ting-Yu Lin, Chih-Hsi Kuo, Fu-Tsai Chung, Pai-Chien Chou, Po-Jui Chang, Yung-Lun Ni, Shu-Chuan Ho, Horng-Chyuan Lin, Chun-Hua Wang, Chih-Teng Yu, Han-Pin Kuo

**Affiliations:** 1 Department of Thoracic Medicine, Chang Gung Memorial Hospital and Chang Gung University, School of Medicine, 199 Tun-Hwa N. Rd., Taipei, Taiwan; 2 Division of Pulmonary, Department of Internal Medicine, Shuang Ho Hospital, Taipei, Taiwan; 3 School of Respiratory Therapy, College of Medicine, Taipei Medical University, Taipei, Taiwan; 4 Department of Chest Medicine, Buddhist Tzu Chi General Hospital, Taichung Branch, Taichung, Taiwan

**Keywords:** Chronic cough, Obstructive sleep apnoea, Continuous positive airway pressure

## Abstract

**Background:**

Obstructive sleep apnoea (OSA) has recently been identified as a possible aetiology for chronic cough. The aim of this study was to compare the incidence of chronic cough between patients with and without OSA and the impact of continuous positive airway pressure (CPAP) treatment in resolving chronic cough.

**Methods:**

Patients referred to the sleep laboratory from January 2012 to June 2012 were retrospectively enrolled. Clinical data, treatment course and resolution of chronic cough were analysed. Specifically, gastro-oesophageal reflux (GERD), upper airway cough syndrome, asthma, apnoea-hypopnoea index and the impact of CPAP treatment on chronic cough were assessed.

**Results:**

A total of 131 patients were reviewed. The incidence of chronic cough in the OSA group was significantly higher than the non-OSA group (39/99 (39.4%) vs. 4/32 (12.5%), p = 0.005). Both GERD and apnoea-hypopnoea index were significantly associated with chronic cough in univariate analysis. After multivariate logistic regression, GERD was the only independent factor for chronic cough. Moreover, the resolution of chronic cough was more significant in the OSA patients with CPAP treatment compared with those not receiving CPAP treatment (12/18 (66.7%) vs. 2/21 (9.5%), p = 0.010).

**Conclusion:**

The incidence of chronic cough was significantly higher in the OSA patients. In addition, CPAP treatment significantly improved chronic cough. Therefore, OSA may be a contributory factor to chronic cough.

## Introduction

The incidence of chronic cough ranges from 9% to 33% of the adult population [1,2]. The most common aetiologies for chronic cough in non-smokers are upper airway cough syndrome (UACS), gastro-oesophageal reflux (GERD) and asthma, all of which are empirically treated [2,3]. However, the aetiologies of 12% to 42% of coughs are unexplained despite thorough evaluation [4]. Therefore, it is important to explore other possible aetiologies for chronic cough. A recent study reported four patients with unexplained chronic cough who were found to have obstructive sleep apnoea (OSA). Moreover, a prospective study also reported that of 108 patients being referred to sleep clinics for sleep disordered breathing, 33% had a co-existing cough [5], which suggests an association between chronic cough and OSA. In addition, another study reported that 44% of patients with chronic cough had OSA, 93% of whom demonstrated a significant improvement in cough with continuous positive airway pressure (CPAP) treatment [6]. The mechanism between chronic cough and OSA is still not clear, although GERD, UACS and airway inflammation have been proposed to be involved [7]. However, studies on these topics have lacked an adequate control group or included a small sample size [5-7]. Therefore, the aim of this study was to evaluate the prevalence of chronic cough and the associated factors in patients with OSA, and the effect of CPAP treatment.

## Materials and methods

### Study population

We retrospectively recruited patients with suspected OSA who were referred to our sleep lab by thoracic doctors from January 2012 to June 2012 in Chang Gung Memorial Hospital, a tertiary hospital in Taiwan. Patients were excluded if they were smokers, or if they had had an acute upper airway infection in the past 4 weeks, abnormal chest X-rays or any history of malignancy. The Chang Gung Medical Foundation Institutional Review Board approved this study (102-2103B) and waived the requirement for informed consent due to the retrospective nature of the study.

### Study design

The medical records of each patient were reviewed to collect the clinical characteristics and laboratory results. In addition, data on comorbidities, aetiologies for the chronic cough such as GERD, UACS and asthma, medications, pulmonary function, Epworth sleepiness scale and clinical follow-up for 3 months after CPAP treatment were analysed. The improvements in cough are decided according to patients’ self-report during following visits.

#### Definitions

A chronic cough was defined as a cough lasting for 2 months or more. Upper airway cough syndrome was defined as: (1) patients describing the sensation of “having something drip down into their throat” and/or the need to frequently clear their throat; (2) computed tomographic imaging or Water’s view showing chronic sinusitis, or a positive finding by nasopharyngoscopy; (3) response to intranasal corticosteroids or anti-histamines [8,9]. Asthma was defined as a positive result of provocation test or PEF variability rate >20% [8]. GERD was defined as a response to anti-GERD medication [8], or if 24-h pH level exceeded the 95th percentile for percentage total time with a pH < 4 of > 4.8% [9]. Sleep stages and arousals were scored according to the AASM criteria [10]. Established criteria were used to score respiratory events such as hypopnea, obstructive apnoea, central apnoea, mixed type apnoea, and Cheyne-Stokes respiration [11]. Apnoea was defined as oronasal flow cessation for more than 10 seconds. Hypopnea was defined as a 50% reduction in oronasal flow for more than 10 seconds or a 30% reduction followed by arousal or more than 3% decrease in SaO2. Based on the polysomnography results, OSA was defined as an apnoea/hypopnea index (AHI) > 15 per hour, of which ≥ 50% were obstructive. CPAP titration to determine the optimal pressure was performed according to standard guidelines [12].

#### Statistical analysis

Data were expressed as mean ± SD (standard deviation) or mean ± SEM (standard error of the mean). The Student’s *t* test was used for comparisons of continuous variables between the two groups, while the Mann–Whitney test was used for non-normal distributions. Categorical variables were compared by chi-square or Fisher’s exact tests. The Pearson product correlation coefficient was used to examine correlations between variables and chronic cough. Multivariate logistic regression analysis was used to determine the independent factors associated with chronic cough. A p value less than 0.05 was considered to be statistically significant. All analyses were performed using the SPSS software package version 13.0 (SPSS Inc., Chicago, IL, USA).

## Results

### Demographic and clinical characteristics of the patients

A total of 147 patients with suspected OSA were identified at our sleep lab between January 2012 and June 2012, 30 of whom were excluded due to the following reasons: 8 (5.4%) were current smokers; 5 (3.4%) had had acute upper airway infections in the past 4 weeks; 2 (1.4%) had abnormal chest X-rays, and 1 (0.7%) had a malignancy. The records of the remaining 131 patients were further reviewed, of whom 99 had OSA and 32 did not. The baseline demographic data and clinical characteristics of these patients are listed in Table 1. The mean ages of the patients with and without OSA were similar (52.2 and 48.3 years, respectively). The mean AHI in the OSA group was 53.6 ± 24.7/h of sleep, indicating that most of the patients had severe OSA, accompanied with a higher percentage of males (75.8%) and higher BMI (28.9 ± 4.1 vs. 24.9±4.3) compared with the Non-OSA group. Moreover, the percentage of chronic cough was significantly higher in the OSA group compared to the Non-OSA group (39.4% vs. 12.5%, p = 0.005). Interestingly, the incidence of GERD was also significantly higher in the OSA group, while the incidence of UACS and asthma was similar between the two groups. Other characteristics including Epworth sleepiness scale, pulmonary function, and medications including nasal steroids, anti-histamines, angiotensin converting enzyme inhibitors, angiotensin receptor blockers, inhaled corticosteroids and long-acting β_2_ agonists were also similar between the two groups.

**Table 1:** Subjects demonstration

**Characteristics**	**OSA** **n = 99**	**Non-OSA** **n = 32**	***p-*** **value**
Age	52.2±11.6	48.3±13.1	0.105
Male, n (%)	75 (75.8)	17 (53.1)	0.025
BMI	28.9±4.1	24.9±4.3	0.000
**Epworth Sleepiness Scale**	12.9±4.5	12.8±5.5	0.884
Total AHI, /h	53.6±24.7	10.1±4.3	0.000
**Pulmonary function test**			
FEV_1_/FVC	83.7±8.4	82±12.5	0.394
FEV_1_ (% predicted)	82.6±20.1	82.6±17.5	0.993
FVC (% predicted)	82.4±17.9	86.8±22.3	0.278
**Chronic cough**	39 (39.4)	4 (12.5)	0.005
**Upper airway cough syndrome**	79 (79.8)	23 (71.9)	0.340
**Gastro-esophageal reflux disease**	43 (43.4)	5 (15.6)	0.006
**Asthma**	18 (18.2)	5 (15.6)	1.000
**Medication**			
Nasal steroid	72 (72.7)	20 (62.5)	0.276
Anti-histamine	79 (79.8)	23 (71.9)	0.340
ACEI or ARB	11 (11.1)	0 (0.0)	0.065
Inhaled corticosteroid	18 (18.2)	5 (15.6)	1.000
Long-acting β_2_ agonist	4 (4.0)	2 (6.2)	0.634
Proton pump inhibitor	43 (43.4)	5 (15.6)	0.006

Data are presented as mean ± SD; *BMI* body mass index, *FEV1* forced expiratory volume in one second, *FVC* forced volume capacity, *ACEI* angiotensin converting enzyme inhibitor, *ARB* angiotensin receptor blocker.

### Univariate and multivariate logistic regression analysis for the variables associated with chronic cough

In univariate analysis, AHI was significantly correlated with chronic cough (Table 2). GERD was also significantly correlated with chronic cough, while UACS, asthma and BMI were not significantly correlated with chronic cough. AHI, UACS, GERD and asthma were then used in the multivariate logistic regression model, which showed that GERD was the only independent factor associated with chronic cough (Table 3).

**Table 2:** Univariate analysis of the variables associated with chronic cough

**Parameter**	**beta**	**Standard error**	**95% CI**	**P value**
BMI	0.060	0.042	0.98 to 1.15	0.153
Male	−0.033	0.406	0.437 to 2.15	0.936
AHI	0.016	0.007	1.00 to 1.03	0.019
GERD	2.379	0.434	4.61 to 25.27	0.000
Upper airway cough syndrome	0.539	0.481	0.67 to 4.40	0.262
Asthma	0.778	0.468	0.87 to 5.44	0.096
FEV1 (%)	−0.005	0.010	0.98 to 1.02	0.650

*BMI* body mass index, *AHI* apnoea-hypopnoea index, *GERD* Gastro-oesophageal reflux disease, *FEV1* forced expiratory volume in one second.

**Table 3:** Multivariate analysis with logistic regression: factors associated with chronic cough

**Factors**	**beta**	**SE**	**P value**	**OR**
AHI	0.009	0.008	0.245	1.009
Upper airway cough syndrome	0.291	0.556	0.601	1.337
GERD	2.339	0.453	0.000	10.373
Asthma	0.884	0.554	0.110	2.422

*AHI* apnoea-hypopnoea index, *GERD* Gastro-oesophageal reflux disease.

### Cough response to CPAP treatment in the patients with OSA and chronic cough

A total of 39 patients with both OSA and chronic cough were identified, of whom 18 (46.2%) received CPAP treatment for 3 months (Table 4). A significant improvement in the chronic cough was noted in the patients who received CPAP treatment compared to those who did not receive CPAP treatment (12/18 (66.7%) vs. 2/21 (9.5%), p = 0.010). In addition, CPAP treatment was also beneficial for the patients with both OSA and asthma, whose chronic cough was significantly improved by CPAP treatment compared to those who did not receive CPAP treatment (3/4 (75%) vs. 1/7 (14.3%), p = 0.044). Similar results were also found in UACS and GERD (2/18 (11.1%) vs. 10/14 (71.4%), p = 0.001; and 1/15 (6.7%) vs. 8/14 (57.1%), p = 0.005, respectively).

**Table 4:** Comparison of the OSA patients with chronic cough who did and did not receive CPAP treatment

**Characteristics**	**OSA with CPAP treatment** **n = 18**	**OSA without CPAP treatment** **n = 21**	***p-*** **value**
Age	49.8±9.5	57.1±10.8	0.055
Male, n (%)	16 (88.9)	14 (66.7)	0.139
BMI	29.2±5.4	29.1±3.4	0.602
**Pulmonary function test**			
FEV_1_/FVC	78.9±12.5	85.3±6.0	0.193
FEV_1_ (% predicted)	76.6±23.5	85.4±16.8	0.317
FVC (% predicted)	79.4±19.5	86.2±16.6	0.367
**Epworth Sleepiness Scale**	12.9±4.1	13.4±5.3	0.618
**Upper airway cough syndrome**	18 (85.7)	14 (77.8)	0.682
**Gastro-esophageal reflux disease**	14 (77.8)	15 (71.4)	0.726
**Asthma**	4 (22.2)	7 (33.3)	0.497
**Sleep parameters**			
Total AHI, /h	59.5±26.4	51.8±27.1	0.353
CPAP titration pressure	8.8±3.0	7.3±1.8	0.318
**Improved cough, n (%)**	12 (66.7)	2 (9.5)	0.010

Data are presented as mean ± SEM.

*BMI* body mass index, *FEV1* forced expiratory volume in one second, *FVC* forced volume capacity, *AHI* apnoea-hypopnoea index.

## Discussion

This study demonstrated that the prevalence of chronic cough was significantly higher in the OSA group. In univariate analysis, AHI and GERD were significantly associated with chronic cough. In multivariate analysis, GERD was the only factor associated with chronic cough, which was significantly improved after CPAP treatment. To the best of our knowledge, this is the first study to report an association between chronic cough and OSA.

The most common etiologies of chronic cough are GERD, rhinosinusitis, and asthma [13]. Recently, several reports have suggested an association between chronic cough and obstructive apnoea [5-7]. Chan et al. reported that the prevalence rate of chronic cough in OSA patients is up to 33% [5], which is much higher than that in the general population [14,15]. In Chan et al’s study [5], both GERD and rhinitis played important roles in chronic cough, however, there was no control group and the number of cases was relatively small. In the present study, the prevalence of chronic cough in the patients with OSA was 38.6%, which is similar to the results of Chan et al. [5]. In addition, the number of cases in the present study was larger, and most importantly, the present study enrolled a control group. Compared to the control group, the incidence of chronic cough was significantly higher in the OSA group. Interestingly, the incidence of GERD was also significantly higher in the OSA group, but not UACS or asthma.

GERD is known to be an important aetiology of chronic cough, and a higher prevalence of GERD is expected in patients with OSA due to large intrathoracic negative pressure swings during apnoea episodes aggravating the severity of GERD [16]. Several sleep lab-based studies have reported incidence rates of GERD in OSA patients ranging from 64.7% to 100% [17-19]. In a large cross-section epidemiology study, subjects with nocturnal GERD had a significantly higher incidence of OSA than those without nocturnal GERD (16% vs. 5%). Nasal CPAP has been shown to reduce GERD in patients with OSA [19,20], suggesting a strong relationship between GERD and OSA. In the present study, only AHI and GERD were associated with chronic cough in univariate analysis, and only GERD was associated with chronic cough in multivariate analysis. This implies that GERD may be the most important aetiology of chronic cough in patients with OSA. However, the present study is a retrospective study, and further large-scale prospective studies are needed to draw a more definitive conclusion.

Nasal obstruction is also associated with OSA, and possible mechanisms such as the Starling resistor model, unstable oral breathing, nasal-ventilatory reflex and nitric oxide have been identified [21]. In the Wisconsin Sleep Study, subjects with self-reported nocturnal nasal congestion had a three-fold increase in the incidence of snoring [22]. On the other hand, a prospective study reported that allergic rhinitis is directly associated with OSA [23]. The use of nasal steroids has been reported to improve sleep quality, but not the severity in patients with severe OSA [24] or in those who receive nasal surgery [25]. Therefore, it is reasonable to assume nasal steroids or surgery does not improve chronic cough, which is related to OSA. In the present study, a high percentage of rhinosinusitis was noted in the OSA patients, and most of them were treated with nasal steroids and anti-histamines while only some with nasal surgery. Further, rhinosinusitis was not associated with chronic cough in the present study.

The incidence of asthma in patients with chronic cough has been reported to range from 16% to 41.8%, and coughing has been reported to be significantly improved by inhaled corticosteroid treatment [26]. However, a significantly higher dose of inhaled corticosteroids is needed to control asthma when sputum coexists with eosinophils and neutrophils [27]. Moreover, neutrophils are activated and delay apoptosis [28,29] during the process of ischemia/reperfusion caused by OSA. Therefore, OSA is an important factor in aggravating asthma control, which can be reversed by CPAP treatment [30]. In addition, the asthma-related chronic cough, which is aggravated by OSA, can also be improved by CPAP. In the present study, chronic cough was significantly improved in the patients with both OSA and asthma by CPAP treatment compared to those who did not receive CPAP treatment (3/4 (75%) vs. 1/7 (14.3%); p = 0.044). However, asthma was not an independent factor contributing to chronic cough in this study, and the number of case was relatively small. Further large-scale studies are needed to clarify this issue.

The major limitations of the present study are its retrospective nature, which may have led to bias in patient selection. Second, the sample size of the study is small, and therefore the results of the study should be interpreted with caution. A prospective study with a larger sample size is warranted to further confirm the results. Finally, the population in this study was based in a sleep lab, so extrapolation of the results to the general population should be done with caution.

## Conclusions

In conclusion, the incidence of chronic cough was significantly higher in the patients with OSA. Both GERD and AHI were significantly associated with chronic cough in univariate analysis, however GERD was the only independent factor associated with chronic cough in multivariate analysis. Chronic cough was significantly improved after CPAP treatment for the patients with OSA, and therefore OSA may be a contributory factor to chronic cough.

## Abbreviations

BMI: Body mass index; FEV1: Forced expiratory volume in one second; FVC: Forced volume capacity; ACEI: Angiotensin converting enzyme inhibitor; ARB: Angiotensin receptor blocker; AHI: Apnoea-hypopnoea index; GERD: Gastro-oesophageal reflux disease; FEV1: Forced expiratory volume in one second; CPAP: Continuous positive airway pressure; UACS: Upper airway cough syndrome.

## Competing interests

The authors declare that they do not have any financial competing interests in relation to the current manuscript.

## Authors’ contributions

T-YW contributed to conceptualization and design of this study; collection, analysis, and interpretation of the data; and preparation of the manuscript. Y-LL contributed to conceptualization and design of this study; collection, analysis, and interpretation of the data; and preparation of the manuscript. T-YW, Y-LL, W-TL, S-ML, T-YL, C-HK, F-TC, P-CC, P-JC, Y-LN, S-CH, H-CL, C-HW, C-TY contributed to collection, analysis, and interpretation of the data and preparation of the manuscript. H-PK contributed to conceptualization and design of the study; collection and interpretation of the data; and preparation of the manuscript. All authors read and approved the final manuscript.
